# New Prototype Screened Doors and Windows for Excluding Mosquitoes from Houses: A Pilot Study in Rural Gambia

**DOI:** 10.4269/ajtmh.18-0660

**Published:** 2018-10-22

**Authors:** Musa Jawara, Ebrima Jatta, David Bell, Thomas R. Burkot, John Bradley, Victoria Hunt, Balla Kandeh, Caroline Jones, Aji Matty Manjang, Margaret Pinder, Shannon Stone, Umberto D’Alessandro, Jakob Knudsen, Steve W. Lindsay

**Affiliations:** 1 Medical Research Council Unit, The Gambia, London School of Hygiene and Tropical Medicine, Fajara, The Gambia;; 2 National Malaria Control Programme, Banjul, The Gambia;; 3 Intellectual Ventures Global Good Fund, Bellevue, Washington;; 4 Australian Institute of Tropical Health and Medicine, James Cook University, Cairns, Australia;; 5 Department of Infectious Diseases, London School of Hygiene and Tropical Medicine, London, United Kingdom;; 6 KEMRI-Wellcome Trust Research Programme, Kilifi, Kenya;; 7 Nuffield Department of Medicine, Centre for Tropical Medicine and Global Health, University of Oxford, Oxford, United Kingdom;; 8 Department of Biosciences, Durham University, Durham, United Kingdom;; 9 The Royal Danish Academy of Fine Arts, School of Architecture, Design and Conservation, The School of Architecture, Copenhagen, Denmark

## Abstract

Despite compelling evidence that modern housing protects against malaria, houses in endemic areas are still commonly porous to mosquitoes. The protective efficacy of four prototype screened doors and two windows designs against mosquito house entry, their impact on indoor climate, as well as their use, durability and acceptability was assessed in a Gambian village. A baseline survey collected data on all the houses and discrete household units, each consisting of a front and back room, were selected and randomly allocated to the study arms. Each prototype self-closing screened door and window was installed in six and 12 units, respectively, with six unaltered units serving as controls. All prototype doors reduced the number of house-entering mosquitoes by 59–77% in comparison with the control houses. The indoor climate of houses with screened doors was similar to control houses. Seventy-nine percentage of door openings at night occurred from dusk to midnight, when malaria vectors begin entering houses. Ten weeks after installation the doors and windows were in good condition, although 38% of doors did not fully self-close and latch (snap shut). The new doors and windows were popular with residents. The prototype door with perforated concertinaed screening was the best performing door because it reduced mosquito entry, remained fully functional, and was preferred by the villagers. Screened doors and windows may be useful tools for reducing vector exposure and keeping areas malaria-free after elimination, when investment in routine vector control becomes difficult to maintain.

## Introduction

Despite the considerable gains made in global malaria control from 2001 to 2012, when malaria mortality fell by 45% in all age groups,^1^ the disease remains a substantial public health problem causing 216 million cases and 445,000 malaria deaths in 2016.^2^ The reduction in malaria was achieved largely by massive deployment of long-lasting insecticidal nets (LLINs) and indoor residual spraying (IRS), together with prompt and effective treatment of human malaria cases. Yet, the future success of these vector control interventions is threatened by poor coverage of LLINs, inconsistent use, and the growing problem of insecticide-resistant mosquitoes,^2,3^ and the continued financial burden of these time-limited interventions. Additional interventions are needed to complement the current insecticide-dependent tools for optimal protection and for use in malaria-elimination areas, when deployment of LLINs and IRS are withdrawn or scaled down.

Because ≥ 80% of malaria transmission in sub-Saharan Africa occurs indoors at night,^4^ preventing mosquitoes from entering houses will reduce transmission further. A recent systematic review and meta-analysis of the literature on housing and malaria found strong evidence that “modern” housing is protective against malaria in many tropical countries.^5^ Overall, residents of more modern homes were 42% less likely to have a malaria infection compared with traditional homes and a 54–65% lower risk of being sick with malaria. The importance of housing in reducing malaria and other vector-borne diseases was well recognized by the World Health Organization (WHO) in their global strategy, the Global Vector Control Response 2017–2030,^6^ and highlighted in the recent guidance “Keeping the vector out: Housing improvements for vector control and sustainable development.”^7^

**Figure 1.**
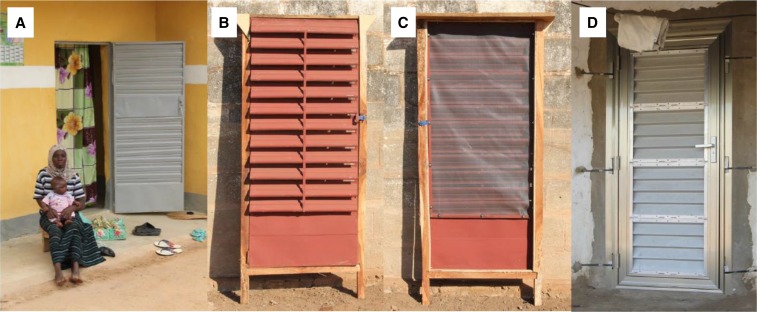
Evolution of the louvered door. (**A**) Typical unscreened Francophone door from West Africa; (**B**) Roo*Pf*s door external view; (**C**) Roo*Pf*s door internal view, showing screening and (**D**), the concertinaed screened door, D1. This figure appears in color at www.ajtmh.org.

There have been two household randomized-controlled studies of house screening^8,9^ with a further study in progress.^10^ In one of those studies, in The Gambia, screened houses had 59% fewer *Anopheles gambiae* s.l., the principal vector of malaria in sub-Saharan Africa, than unscreened houses, while in a study in Ethiopia, there were 48% fewer *Anopheles arabiensis* indoors in screened houses compared with unscreened ones.^11^ In the Gambian study, the wooden door was modeled on a design recommended by the WHO,^12,13^ based on a door originally used by the Tennessee Valley Authority in 1947 in the United States.^14^ In a more recent study in The Gambia, a new type of screened door based on a metal-louvered door common in Francophone West Africa (the Roo*Pf*s door) was evaluated (Figure 1).^10^ The Roo*Pf*s door was constructed with fixed metal louvers and internal mosquito netting to prevent mosquitoes entering the house. A bungee cord with hooks from the Roo*Pf*s door to the frame closed the door automatically. After 6–12 months, however, many of the doors were warped, with damaged screening, and failed to close automatically. Attributes of the Roo*Pf*s door (self-closing, mosquito-excluding, and screening) were integrated in a novel design made entirely of metal; the concertina door.

In many parts of sub-Saharan Africa, houses have closed eaves and the only entry points for mosquitoes are around badly fitting doors and windows. Screened and well-fitted doors and windows in houses were hypothesized as a means to reduce the entry of mosquitoes at night and increase air ventilation to keep the house cool at night. A pilot study in a Gambian village compared houses fitted with new designs of doors and windows with unaltered houses for their protective efficacy against house-entering mosquitoes, indoor climate, door opening and closing, short-term durability of the doors and windows, and the acceptability of the prototypes designs. The pilot study was designed to guide product development and provide data for designing an epidemiological intervention study using screened doors and windows.

## Methods

### Study site.

The study site was in Wellingara village (N 13°33.365′, W 14°55.461′) on the south bank of the River Gambia in Lower Fulladu West, Central River Region, in The Gambia, during the rainy season, from the end of August until the end of November in 2017. Wellingara village has a population of 629 people,^15^ mostly of Mandinka ethnicity (90%), and is situated in an area of extensive irrigated rice cultivation in open Sudan savanna.^16^ All 99 dwelling houses in the village were single-story and ranged in size from 1 to 24 rooms.

**Figure 2.**
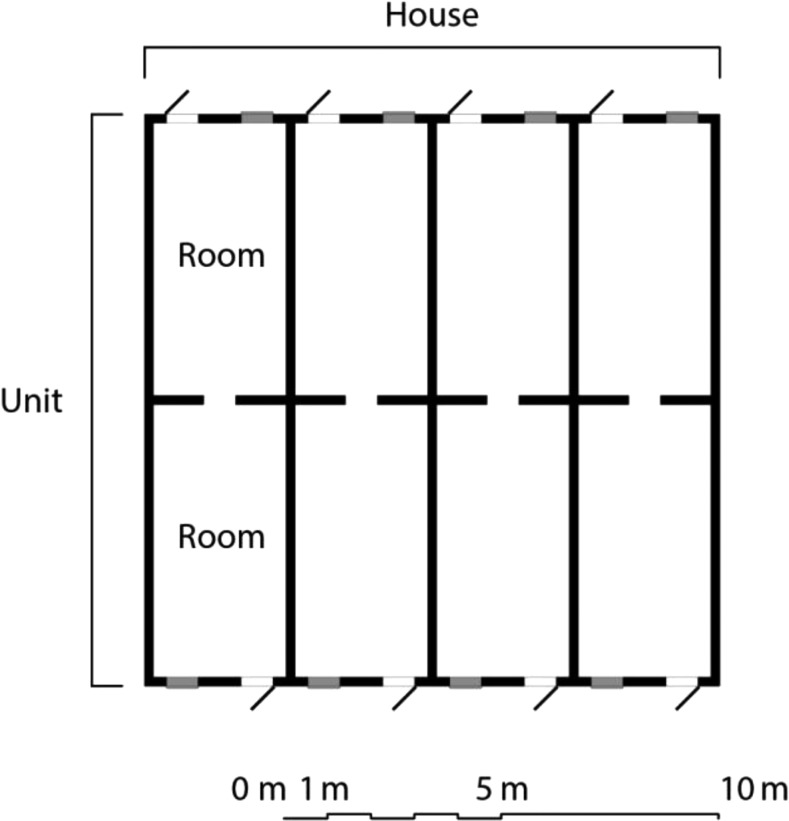
A stylized plan view of a Gambian line house, showing the separate units, each consisting of two rooms.

Village houses were mostly line houses, with several similar sized and structured units built longitudinally (a terraced housing design),^17^ with a metal roof and mud block walls (Figure 2, Table 1). Units have two adjoining rooms separated by an approximately 2-m high wall in the center where the roof is at maximum height. The doors are of a standard size, the height and width of a single sheet of locally sourced corrugate roofing (70 × 180 cm). Typically, children and women occupy separate units from adult men. Bednet coverage is high in the area and more than 90% of the population reported sleeping under a net throughout the year. The National Malaria Control Programme conducted IRS campaigns in the village, with bendiocarb, a carbamate insecticide, between October 1 and October 15, 2016, and with Actellic, an organophosphate insecticide, in August 2017, immediately before the installation of the doors and windows. Twenty-seven of the 30 units in the pilot study were sprayed with Actellic.

**Table 1:** Characteristics of housing units (*n* = 30) in Wellingara village immediately before door and window installation

Characteristic	Measurement
Number of housing units	30
Roof type	30 metal (100%)
Wall type	30 mud blocks (100%)
Unit position	16 at the end of line house (53%), 14 in the middle (47%)
No. of adults	1
No. of children	1 (0–3)
No. of beds	1
No. of long-lasting insecticidal nets	1
Room width (m)	3.06 (3.00–3.26)
Room length (m)	6.28 (6.15–6.46)
Room height in centre (m)	3.65 (3.54–3.80)
Back wall height (m)	2.55 (2.49–2.59)
Centre wall height (m)	2.29 (2.23–2.37)
Interior air volume (m^3^)	61.37 (54.99–65.39)
Air volume/person (m^3^)	22.43 (14.02–34.62)
No. units sprayed with Actellic insecticide	27 (90%)

Data are totals and medians. Values in parentheses are percentages or 95% confidence limits.

### Study design.

The village was mapped in February 2017, following approval by the Alkalo (village chief) and other village opinion leaders. There were 74 suitable units in the village, of which 37 randomly selected, stratified by house, with a maximum of two units/house and then randomly allocated to the five study arms, with six units/arm. In selected units, house owners were recruited to the study, if they consented individually to a field assistant. In each enrolled unit, any gaps in the walls, eaves, or between adjacent rooms were closed with mortar and mud blocks, making mosquito house entry possible only through the doors or windows.

### Interventions.

The intervention was screened doors and windows (Table 2) designed to prevent mosquito entry, to provide security and privacy and to increase airflow to the house. The designs of the doors and windows were based on target product profiles (TPPs, Supplemental Materials 1 and 2) developed by a panel of vector biologists, architects, engineers, and members of nongovernmental organizations working to reduce malaria transmission.

**Table 2:** Study groups

Group	Door type	Window type	No. of housing units in each group
Control	Local	Local	6
1	D1	W1	3
2	D1	W2	3
3	D2	W1	3
4	D2	W2	3
5	D3	W1	3
6	D3	W2	3
7	D4	W1	3
8	D4	W2	3

Where D refers to the type of prototype door and W is the type of prototype window.

**Figure 3.**
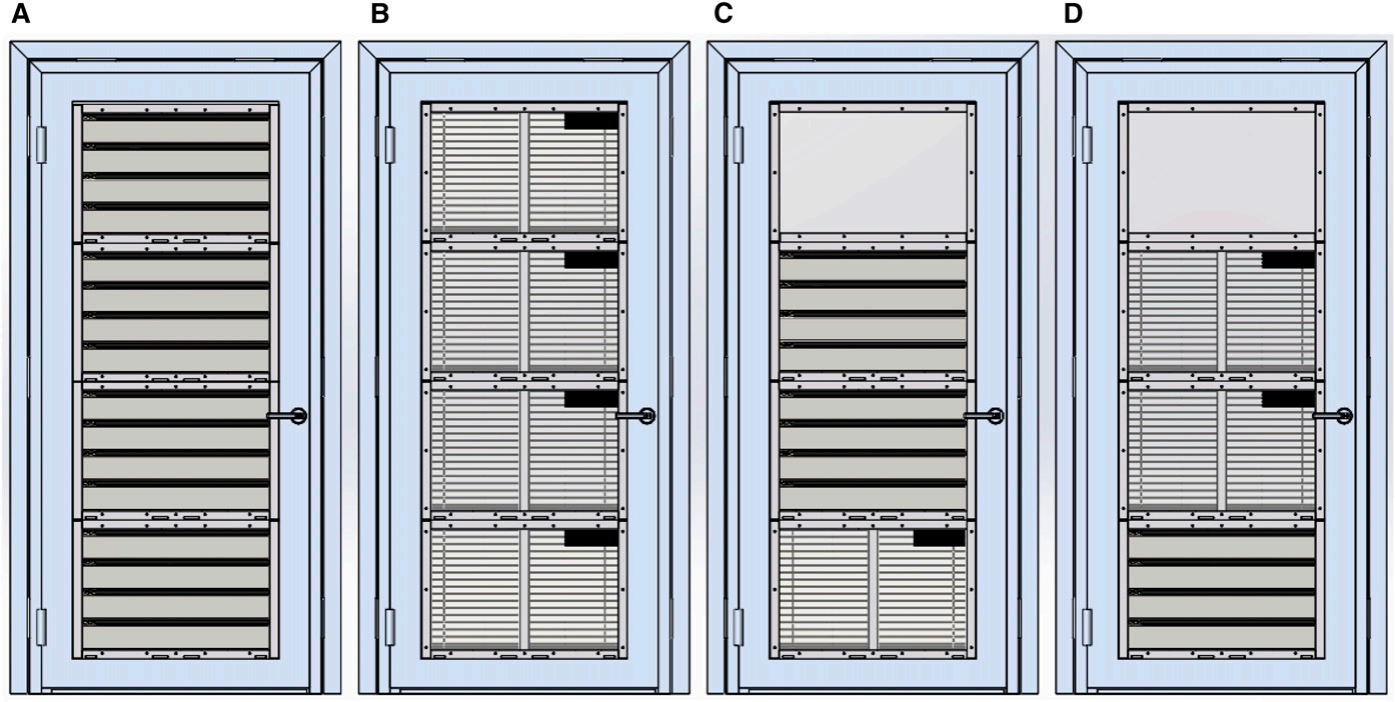
Prototype doors (internal view). Where **A** is the concertinaed door (D1), **B** is the blinds door (D2), and **C** and **D** are doors with a combination of panels and opaque windows at the top (D3 and D4). This figure appears in color at www.ajtmh.org.

**Figure 4.**
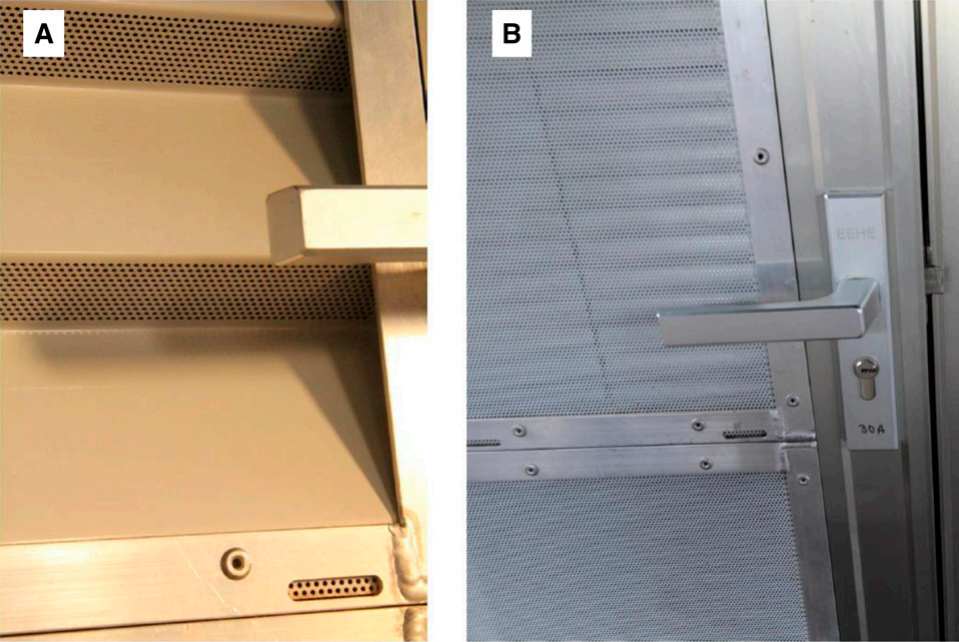
Details of (**A**), the concertinaed door (external view) (D1) showing perforations underneath the folded metal, and (**B**), the blinds door (external view) (D2), where the outer surface is perforated and venetian blinds are on the inner surface. This figure appears in color at www.ajtmh.org.

**Figure 5.**
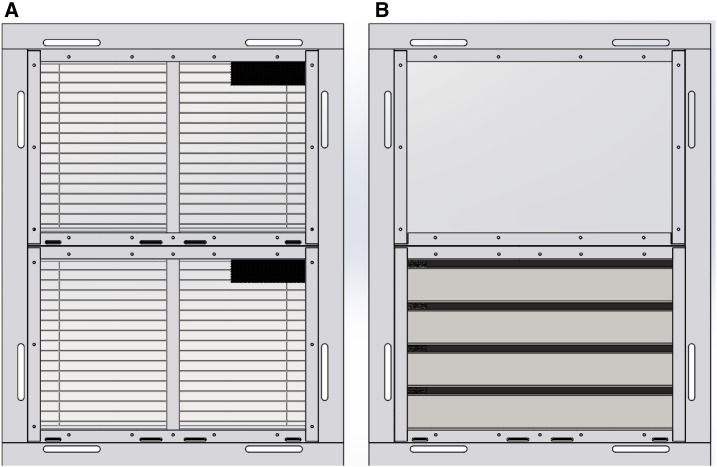
Prototype windows (internal view). Where **A** is the blinds window (W1) and **B** is a window combining an opaque panel at the top and concertinaed panel at the bottom (W2). This figure appears in color at www.ajtmh.org.

Based on the TPPs, four new state-of-the-art screened door and two window designs using a modular system were developed. Windows and doors were constructed using the same modules, held in place with an aluminum frame. Each doorframe contained four modules and each window two modules. There were four metal self-closing prototype doors (Figure 3). Like the door in the Roo*Pf*s trial, the “concertina door” (D1) had metal louvers. Although the upper metal surfaces were solid, to provide privacy and rain protection, the lower surfaces (parallel to the ground) were perforated with 1.61 mm diameter holes, to allow airflow but not mosquito entry (Figure 4A). The “blinds door” (D2) consisted of perforated metal panels on the external surface and venetian blinds internally (Figure 4B). Two prototypes (D3 and D4) integrated the features of the concertinaed and blind doors with a translucent window to provide both privacy and light. D3 had a translucent panel at the top, with two concertinaed panels below and a blinds panel at the bottom. D4 had a translucent panel at the top, followed by two blinds panels and a concertinaed panel at the bottom. Each door opened outward, to push mosquitoes away from the room, and was self-closing by three spring hinges and lockable. There were two window prototype designs tested (W1 and W2; Figure 5). W1 had two blinds panels and W2 had a translucent panel at the top and a concertinaed panel at the bottom. Both windows were designed to provide light, privacy, and air through the concertinaed areas.

Local masons removed the original doors and windows and enlarged the door and window openings to accommodate the aluminum frames (outer dimensions, 188.4 cm high, 93.9 cm wide, and 11 cm deep) required to hold the prototype doors and windows. Prototype doors were 175 cm high, 74.7 cm wide, and 11 cm deep, whereas prototype windows were 90.2 cm high, 73.6 cm wide, and 5.72 cm deep. Doorframes were secured by drilling at least two holes approximately 25 mm in diameter, 250 mm deep, into the mud walls on either side of the vertical frame and filling the holes with wet cement. The frame was then fixed to the walls using 12.7-mm diameter screws, 152.4 mm long, into the wet cement-filled holes. In addition, four holes were drilled externally through the mud blocks and the frame secured with plastic binding tape (Uline Polyester 1.27 cm × 0.4 mm). The frame was then secured in the door gap using cement and the owners vacated their homes for 3 days while the cement cured. The process of door installation is shown in the accompanying video (https://vimeo.com/238767696).

### Entomology.

In previous work, baseline light trap collections were made to help estimate the sample size required for an intervention study in 10 randomly selected rooms one night each week for 5 weeks, from September 28 to the October 24, during the rainy season in 2016. During the present study, collections were made in each unit for eight consecutive weeks from the beginning of September to the end of October 2017. Mosquitoes were sampled using a Center for Disease Control light trap positioned with the light 1 m above the floor, at the foot of the bed, in the back room nearest the back door. Traps were set in the early evening and collected at 07.00 hours the following morning. Mosquitoes were first identified morphologically, with species confirmation for members of the *An. gambiae* complex by polymerase chain reaction (PCR).^18,19^

### Environmental measurements.

The dimensions of each unit, and the number and age of the occupants were recorded. Indoor temperature and relative humidity was measured every 30 minutes using Tinytag data loggers (model, TGU 4500; Gemini Data Loggers, Chichester, United Kingdom) positioned 1 m above the floor on the back wall, of the unit furthest from the bed. Door opening and closing was recorded with a different type of data logger (Onset Hobo, 1-800-data loggers, UX90-001M state/pulse/event/runtime), which were installed at the top of the front and back doors of each unit for four consecutive nights September 28 to the October 24. The condition of the doors and windows was assessed 10 weeks after installation.

### Acceptability assessments.

Focus group discussions (FGDs) with purposively selected householders in each of the five door groups were conducted to explore perceptions of the interventions, including identification of features that were particularly valued or disliked, and the reasons for these preferences. Separate group discussions were held with men and women in each study group, and the advantages and disadvantages of the doors and windows discussed. A minimum of five participants took part in each of the 10 groups (i.e., separate groups of men and women from each of the five door groups). The FGDs were conducted in Mandinka, the local language, by a skilled moderator and with the consent of the participants, the discussions were digitally recorded. The recordings were transcribed and translated into English and written summaries of main points were made.

### Statistical analysis.

The effect of door and window prototype on mosquito house entry was assessed using generalized estimating equations, to adjust for repeat measures, and using a negative binomial model with a logit link function for count data, including door and window type and week in the model. Comparisons of temperature and humidity indoors were made using linear modeling on weekly mean values, adjusting for week. These analyses were based on data recorded at 03.00 hours, during the middle of the normal sleep period, at 16.30 hours, at the hottest time of the day, and at 21.00 hours, when most people go to bed. Comparisons between mosquito counts and the frequency at which doors were opened between 19.00 hours and midnight, the period when mosquitoes begin entering houses, was assessed using linear regression. The software package LadyBug (LadyBug Products, Athol, ID) was used to estimate the percentage of time occupants of various house typologies spent in the “comfort zone.”^20^ The “comfort zone” is defined by the comfort polygon for temperature and relative humidity and provides an estimated percentage of people satisfied with the indoor climatic comfort. The human energy balance model used by the psychrometric chart is the predicted mean vote model developed by Fanger.^21^ In the analysis, we assumed that from 21.00 to 00.00 hours people were seated and quiet, and wore thin straight trousers, briefs, and tee shirts,^22^ and from 00.00 to 06.00 hours people were reclining with the same clothes. Wind speed and radiant temperature were not included. For each typology, the percentage of time the indoor climate was in the “comfort zone” was calculated for two periods: from 21.00 to 00.00 hours, when people retire to bed, and from 00.00 to 06.00 hours, when people are usually sleeping. The duration of door opening was calculated for each hour of the day, excluding 0.2% of occasions when doors had been opened for longer than 1 hour. Comparisons between the frequency of door opening and mosquito abundance were made on nights that light trap collections were made in the unit. Analyses, apart from psychrometric analysis, were carried out using IBM SPSS Statistics 20 (IBM, Armonk, NY) and STATA version 14 (StataCorp LLC, College Station, TX).

### Ethics.

The pilot study was approved by the Gambia Government/Medical Research Council’s joint ethics committee (SCC 1478v3.1, March 16, 2017) and the Department of Biosciences ethics committee, Durham University, United Kingdom (June 29, 2017).

## Results

**Figure 6.**
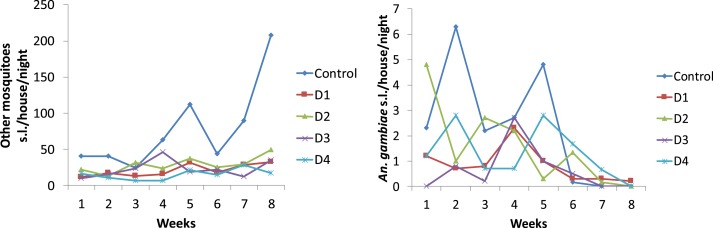
Entry of (**A**), nuisance mosquitoes and (**B**), *Anopheles gambiae* s.l. into houses with different types of doors. This figure appears in color at www.ajtmh.org.

In the study, the predominant mosquitoes collected were culicines with *Mansonia uniformis* and *Mansonia africana* being the most common. During the baseline period in 2016, before the IRS, there were 8.4 *An. gambiae* s.l. (standard deviation [SD] = 11.3) and 64.6 (SD = 64.74) other mosquitoes collected per unit each night. From a sample of 285 *An. gambiae* s.l., 47.4% were identified by PCR as *Anopheles coluzzii* and 52.6% *An. arabiensis*. The numbers of *An. gambiae* s.l. correlated with the number of other species of mosquitoes collected indoors during the first 5 weeks of the study (linear regression on natural log transformed counts, *n* = 150, *F* = 45.3, *R*^2^ = 0.229, *P* < 0.001), suggesting that the numbers of other mosquito species could serve as a proxy for *An. gambiae* s.l. numbers, although this relationship is weak. In the pilot study in 2017, shortly after most houses had been sprayed with insecticide, there were 2.3 *An. gambiae* s.l. (SD = 4.5) and 77.5 other mosquitoes collected per unit each night (SD = 77.3) in unscreened control units. Thus, although the number of other mosquitoes were similar in both years (Mann-Whitney *U* test, *z* = −1.1, *P* = 0.313), there were markedly fewer *An. gambiae* s.l. in 2017 than in 2016 (Mann–Whitney *U* test, *z* = −2.44, *P* = 0.015). In 2017, mosquito numbers, excluding *An. gambiae* s.l., in the control units rose gradually during the study, although this was not discernible in the experimental arms (Figure 6) with the number of *An. gambiae* s.l. remaining low throughout the study, particularly in the last 3 weeks of the study.

All prototype doors reduced the numbers of mosquitoes inside units by 61–79% (Table 3). Fewer *An. gambiae* s.l. were collected from units with prototype doors, but this reduction was not statistically significant after 8 weeks, when mosquito numbers had collapsed. D1 was statistically protective against *An. gambiae* s.l. during the first 5 weeks of the study when *An. gambiae* s.l. populations were highest. There was no difference in mosquito house entry associated with different types of windows.

**Table 3:** The protective efficacy of the different doors prototypes against house-entering mosquitoes

Door prototype	All mosquitoes		*Anopheles gambiae* s.l.			
			Weeks 1–5		Weeks 1–8	
	Rate ratio (95% CIs)	*P*	Rate ratio (95% CIs)	*P*	Rate ratio (95% CIs)	*P*
Control	1		1		1	
D1	0.29 (0.19–0.45)	< 0.001	0.33 (0.14–0.78)	0.011	0.44 (0.18–1.05)	0.064
D2	0.41 (0.25–0.67)	< 0.001	0.66 (0.25–1.76)	0.407	0.83 (0.33–2.06)	0.680
D3	0.33 (0.17–0.64)	0.001	0.36 (0.11–1.13)	0.081	0.43 (0.14–1.32)	0.139
D4	0.23 (0.15–0.35)	< 0.001	0.45 (0.18–1.17)	0.453	0.43 (0.31–1.65)	0.434

CI = confidence interval.

### Environmental measurements.

**Figure 7.**
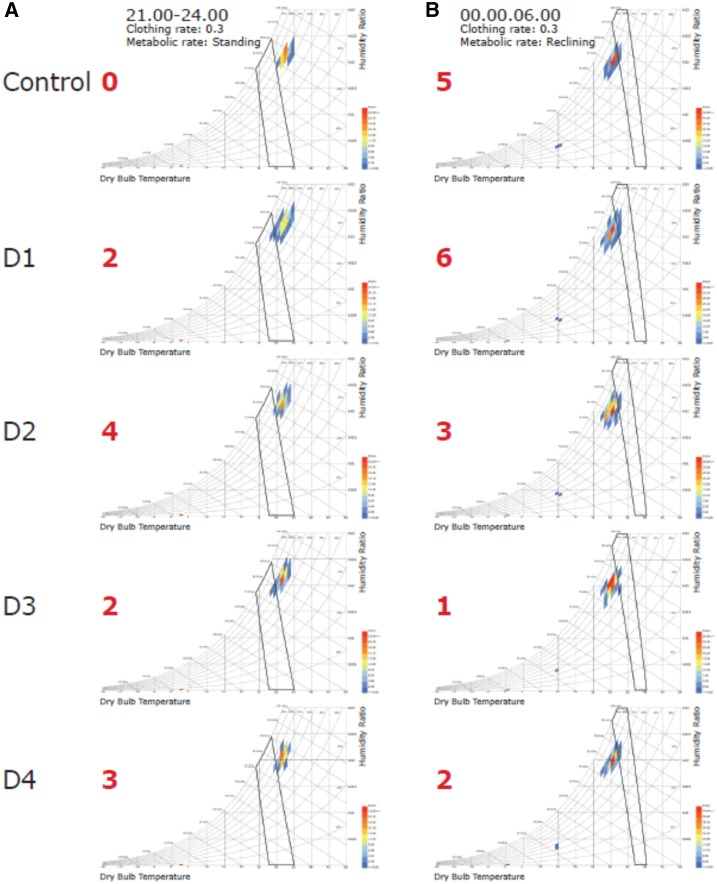
Psychrometric chart. (**A**) Human comfort index from 21.00 hours until 00.00 hours, (**B**) from 00.00 hours to 06.00 hours. Values in red are the percentage of readings that are comfortable. Black polygon indicates comfort area. From 21.00 until midnight all houses are uncomfortably hot, whereas 00.00 to 06.00 hours they are uncomfortably cold, unless using a sheet or blanket. This figure appears in color at www.ajtmh.org.

Indoor temperatures were similar among units fitted with the different prototype doors and windows before and after midnight (Table 4). Moreover, there was no significant difference in indoor temperatures between control and intervention units. The psychrometric analysis shows that all control and intervention units were almost entirely outside the human comfort index for most the night, being too hot before midnight and too cold after midnight (Figure 7). Again, evidence to suggest that the indoor climate differed between control and intervention units was not found.

**Table 4:** Indoor temperature and different door and window types

Group	21.00–00.00 hours		00.00–06.00 hours	
	Mean temperature, °C (95% CIs)	Mean relative humidity, % (95% CIs)	Mean temperature, °C (95% CI)	Mean relative humidity, % (95% CIs)
Comparison between prototype doors				
Door 1	31.5 (31.0, 32.0)	73.6 (71.2, 76.0)	30.0 (29.6, 30.3)	77.3 (74.5, 80.0)
Door 2	31.9 (31.4, 32.4)	74.0 (71.4, 76.6)	30.1 (29.7, 30.5)	76.0 (73.5, 78.5)
Door 3	31.7 (31.2, 32.1)	71.8 (69.6, 74.0)	30.1 (29.8, 30.4)	73.7 (71.5, 76.0)
Door 4	31.8 (31.2, 32.5)	69.1 (65.8, 72.3)	30.5 (30.0, 31.0)	70.6 (67.2, 74.0)
*P*-value	0.558	0.252	0.427	0.088
Comparison between prototype windows				
Window 1	31.5 (31.2, 31.8)	72.5 (70.9, 74.1)	30.1 (29.8, 30.3)	74.5 (72.9, 76.2)
Window 2	32.0 (31.6, 32.3)	71.8 (70.0, 73.6)	30.2 (30.0, 30.5)	74.4 (72.5, 76.2)
*P*-value	0.081	0.595	0.470	0.903
Comparison between control and all prototype doors and windows				
Control	31.7 (31.5, 32.0)	71.9 (70.9, 72.9)	30.2 (30.0, 30.4)	74.4 (73.3, 75.5)
Experimental	32.0 (31.5, 32.5)	70.2 (68.1, 72.4)	30.3 (29.9, 30.7)	73.3 (70.9, 75.8)
*P*-value	0.338	0.187	0.660	0.465

**Figure 8.**
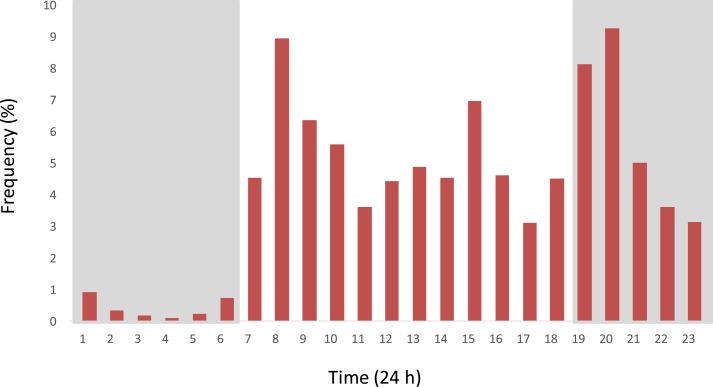
Frequency of door openings during the day (*n* = 15,027 openings in 30 houses over 4 days). This figure appears in color at www.ajtmh.org.

**Figure 9.**
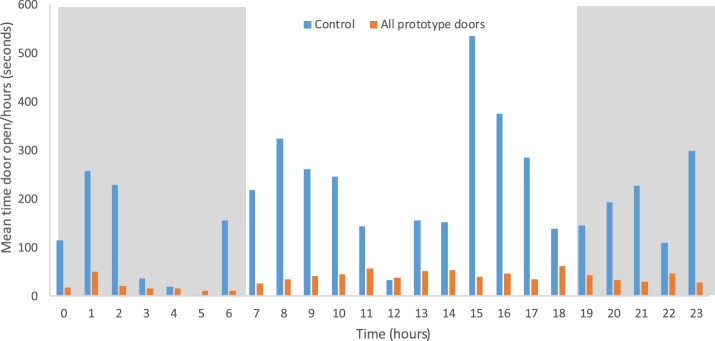
Duration of door opening during the day in control and intervention housing units. This figure appears in color at www.ajtmh.org.

Overall, 79% of door openings at night occurred from 19.00 to 23.59 hours, with only 21% of door openings from 00.00 to 06.00 hours. Activity rose sharply after dawn, followed by two further peaks; one midafternoon and one in the early evening, before gradually declining after 20.00 hours (Figure 8). Duration of door opening was considerably less in the units with prototype doors compared with control houses (paired *t* test, *t* = 6.54, *P* < 0.001; Figure 9). As expected, the numbers of mosquitoes collected indoors increased the more often doors were opened between 19.00 and 00.00 hours (linear regression, *R*^2^ = 0.24, *F* = 10.22, *P* = 0.003), and this relationship was even stronger when only prototype doors were analyzed and control units excluded (linear regression, *R*^2^ = 0.52, *F* = 25.81, *P* < 0.001).

There was evidence that participants highly valued the new doors and windows. Before installation, only four of 20 units had single adult male occupancy, whereas 10 weeks later eight of the units had a single adult male occupant, suggesting that the men were moving into the improved houses, replacing women and children. By the second survey 10 units had hung cotton curtains inside the doors and windows, one had put down new floor covering, one had cemented the floor, one had cemented the floor and walls, and one had covered the walls with decorative cloth. All prototype doors and windows were clean at inspection, except for those belonging to young men, which were dusty.

Structurally the doors and windows frames remained secure to the walls at the conclusion of the study, although hairlike cracks were apparent in some concreting around the door, suggesting that installation strategies may need evaluation for longer term studies. None of the 48 doorframes and doors were dented during the study. There was, however, some damage to a few hinges, with three top hinges having lost inserts and pins, with occasional slight rusting of pins in back door hinges exposed to rain. Front doors were unaffected because they were protected from rain by the overhanging roof. One challenge to mosquito exclusion was that many doors did not automatically close completely, with a small gap of 1–2 mm between door and frame (*n* = 15/48)—probably too small for a mosquito to enter. Another issue was that some of the latch bolts did not fit the rectangular hole in the strike plate in the door frame (*n* = 4/48). Most units had the blinds on doors still intact at the end of the study (front door blinds 34/41 and back door blinds 35/41) and windows (front window 21/24, back window 20/24). Most blinds were left closed, restricting airflow through the door.

### Acceptability.

In most FGDs, the most popular door was the concertinaed door (D1), followed by D3, D4, and D2, whereas for windows, W2 was preferred to W1. Across all FGDs, the participants mentioned that the doors provided privacy, kept out mosquitoes and were attractive to look at.

“the doors are secure and protect the safety and privacy of the dwellers….” (P4 FGD 4)

“mosquitoes and other insects [like flies] hardly enter especially when you take good care of your house.” (P3 FGD 1)

In general, participants thought the doors were attractive, as illustrated by one person who said:

“it is very beautiful and attractive, even passers-by stop to look at it which has raised our status.” (P2 FGD 2)

The participants in most of the FGDs also liked the new features of the prototype door including the ventilated holes and the self-closing door. For example, respondents commented [that]:

“when inside the house, you can see people outside but they do not see you,” (P3 FGD 2)

“the doors are well ventilated because of the holes in it especially in Door 1 and Door 2,” (P6 FGD 4)

and “the door closes behind you without you pulling to close it.” (P6 FGD 4)

The only disadvantage raised about the doors during the FGDs was that doors with blinds could be damaged by small children. One respondent explained that:

“the reason why I don’t like the type of door I have [D2] is simply because I have a lot of children. The children do keep on pulling the venetian blinds down to the ground which spoils it.” (P1 FGD 2)

In most FGDs, respondents liked the windows because they allowed ventilation and looked attractive. Respondents noticed that “fresh air enters the house due to the holes” and it is “beautiful just like the doors.” In some FGDs, people were concerned about the windows not opening:

“The windows are good, but the only problem with them is that it does not open.” (P1 FGD 1)

“The major problem with the windows is their lack of opening but had it been they open and close it would be the best.” (P1 FGD 1)

## Discussion

In this study, the prototype doors and windows reduced the number of house-entering mosquitoes, excluding *An. gambiae*, by 59–77% compared with units by traditional doors and windows. The inability to demonstrate protection against malaria vectors for all door types reflects the extremely low numbers of *An. gambiae* during the study in 2017, due to the spraying of Actellic as an indoor residual spray applied by the National Malaria Control Programme in the village. Recent studies in The Gambia showed that Actellic would have provided protection for the duration of the 10-week study period.^23^

One additional factor contributing to the decline in *An. gambiae* s.l. in the last 3 weeks of the study in 2017 may have been irrigated rice production, the major aquatic habitat for this vector in this area. An earlier study in the neighboring village of Saruja, showed that the numbers of *An. gambiae* s.l. decline as the rice grows and eventually spreads out across the paddies,^16^ unlike *Mansonia* spp., whose numbers were not associated with the stages of rice production but were common during irrigation.^24^ Hence, the sharp decline in *An. gambiae* s.l. in the last 3 weeks of the study may be due to the local mature rice plants making the paddies unsuitable for the vector. The number of “other mosquitoes,” however, was correlated with numbers of *An. gambiae* s.l. If the number of “other mosquitoes” could be used as a proxy for malaria vectors, the evidence suggests that these screened prototype doors and windows can reduce the number of malaria vectors entering houses.

The study units were of a similar size, reducing the variability in attractiveness to mosquitoes, making these ideal for our study. The indoor climate of units fitted with prototype doors and windows were similar to those of normal village units with poorly fitting doors and windows, suggesting that despite the close fit of the doors and windows to their frames, the ventilation engineered into the prototype doors and windows enable airflow comparable with the control houses with poorly fitted doors. The psychrometric charts suggest that control and intervention units were too hot and humid for human comfort before midnight, but after midnight, they cooled rapidly. In practice, sleeping under a bednet and a sheet would make the environment comfortable after midnight. It also presumably explains why there is so little door opening and closing at this time of the night, with most people asleep in bed. Clearly, screened doors and windows are a good start to increase ventilation indoors but additional measures are required to increase the comfort of homes before midnight. This is important because the principal reason for people not sleeping under a bednet is that it is too hot.^25^

The data describing doors opening and closing is, to our knowledge, the first time this behavior has been described in rural sub-Saharan Africa. It reveals a surprising amount of nocturnal activity, particularly, from dusk to midnight when mosquitoes begin entering houses. These results capture the daily activity of village people in this part of The Gambia as witnessed by members of the research team. Most people leave their house shortly after dawn, and there follows a busy period where women sweep the room and men and women prepare for the day. Thereafter, the activity fluctuates throughout the day with a decline in the late afternoon, the hottest part of the day, when people typically rest outdoors where it is cooler. Before midnight there is much movement in and out of the house for several reasons. First, in this Muslim community evening prayers are normally conducted outside at 19.30 hours and 20.45 hours and often require collecting belongings from the house. Second, the evening meal is held outdoors just after or before evening prayers and can involve collecting items from the house such as food bowls and cutlery. Third, water storage jars (Jibadahs) are placed indoors and family members will make repeated trips indoors to collect water with a 1-L drinking cup. Last, young children will spend the first part of the evening sleeping outside on their mothers’ lap or on a mat and are taken indoors, one by one, usually around 22.00 hours,^26^ before normal bedtime for the adults. When it is time for the adults to retire to bed they would normally carry the children outside to use the toilet. Door opening and closing declines slowly, with little activity after midnight. The first call for prayers is made at 5.15 hours and then again at 5.40 hours, with prayers held at 5.45 hours. Thus, some adults will be outside of their LLINs when vectors are still biting shortly before dawn. In our experiments, the duration of door openings was extremely variable, even from day to day, with most doors opening for a few seconds, whereas some doors are kept open for several hours. Our findings show that generally the self-closing prototype doors are open for much shorter periods than control doors, although after 06.00 hours, with the prototype doors, some doors are propped open to allow housework to take place unimpeded.

There is ample evidence that the doors were well appreciated by homeowners. A few older men moved into units with prototype doors and windows, replacing the women and children who had lived there previously. Clearly, when installing doors and windows on a large scale it would be important to maximize the number of prototype doors and windows fitted to provide protection for all family members. Many of the units were beautified with new floor and wall linings and the hanging of curtains behind the doors and windows. Ten weeks after installation most of the doors were clean and tidy, further illustrating that householders valued the prototype doors and windows. The responses from the FGDs confirmed that many villagers prized the doors and windows highly and considered that they were both functional and beautiful. For most, the simple concertina door was preferred, with or without a window module, whereas people were less enthusiastic about doors with blinds, some of which were damaged by small children. For the windows the same was broadly true, with respondents preferring the concertina window, rather than that constructed with blinds.

Structurally, after 10 weeks use, the doors and windows remained intact and in good condition. The frames were still tightly secured to the walls and there was no significant cracking around the door. After 10 weeks, 15% of the blinds were missing and those that remained were probably not adjusted or adjusted very little. Having closed blinds with cloth curtains would have further restricted airflow through the doors and windows, suggesting that the occupants were more concerned with privacy and reducing dust from outside than increasing airflow. The common use of curtains over doors and windows suggest that other screened openings, such as screened eave tubes or screened air bricks need to be considered as an alternative to increase ventilation indoors. Overall, most doors fitted the frame well enough to prevent access of mosquitoes around the doorframe.

The main conclusion from this study is that all prototype doors and windows were effective at reducing the numbers of house-entering mosquitoes. Their effectiveness was probably because of the robust screening and well-fitted doors and windows, with doors that were self-closing, which resulted in fewer opportunities for mosquitoes to enter each unit. The doors and windows were highly desirable and the community was enthusiastic about them. The simple concertina door was preferred by homeowners and it reduced the numbers of *An. gambiae* s.l. and other mosquitoes inside homes. In the future, constructing a window that opens should be investigated, although these would need to self-close. Because there is a tradition of people in rural Gambia having curtains behind the doors and windows, future initiatives need to consider additional ways of increasing indoor ventilation to keep householders cool at night. Where transmission occurs inside houses, new types of screened doors and windows represent a potential supplementary measure for the control of malaria and other vector-borne diseases (where transmission occurs in the house) across sub-Saharan Africa and other tropical regions.

## Supplementary Material

Supplemental figure
