# Supplementary material for: New Prototype Screened Doors and Windows for Excluding Mosquitoes from Houses: A Pilot Study in Rural Gambia
Source: Am J Trop Med Hyg. 2018 Oct 22;99(6):1475–84. doi: 10.4269/ajtmh.18-0660 (PMC6283509; doi:10.4269/ajtmh.18-0660)
Supplement: Supplementary file 1 [file tpmd180660.SD1.pdf]

Target Product Profile: Windows for Mosquito Exclusion	
Characteristic	Response
Key assumptions	There is a growing market in Africa for modern houses.<br><br>Home-owners are investing in house improvement products.<br><br>Home owners will invest in better quality windows that prevent mosquito entry if they are competitive in price with existing products<br><br>Modern houses are associated with reduced malaria transmission in Africa<br><br>Screened windows will reduce mosquito house entry while increasing airflow indoors to keep the house cool<br><br>Increased comfort resulting from more air circulation will enhance mosquito net use.
Rationale	To provide security whilst protecting people from mosquitoes indoors and to help ventilate the house to keep the house cool and increase LLIN use
Goal of the device	Minimum requirements are for the window to exclude mosquito entry, provide security (robust to forced entry), privacy and to maximize air flow.
Target population	sub-Saharan Africa & potentially other tropical and sub-tropical countries
Setting	Rural and urban
Operational price/ item	<$0.50/yr/window assuming an effective life>10 yrs<br><br>Note: final costing dependent on how much does a door cost in Africa from market analyses
Specifications	Should last >10 years as determined from accelerated lifecycle testing<br><br>Shall meet ISO C3 corrosion standard.<br><br>Designed for installation by semi-skilled workers.<br><br>Shall meet water-resistant IPX-2 standard i.e. protecting against rain water when driving from up to 15 degrees from the vertical (driving rain storm). Shall not weigh more than 10 Kg/m2.<br><br>Capable of withstanding “knife shear test” of a trimming knife is pulled x3 along screening with a force of up to 350N (35kg) and a constant downward pressure of 150N (15kg) for a distance of 250mm (https://crimsafe.com.au/testing/)<br><br>Frame compatible with installation of curtains (privacy) and window bars (security)<br><br>Easy to clean
Compatibility	To be used with long-lasting insecticidal nets

Waste disposal	Should be recyclable. At end of life all materials must be disposable.
Drop requirement	n/a
Safety	Should be safe to users – should be designed to not cause injury under normal use.
Benefit over existing methods	Contains no insecticides and could be used as an insecticide-resistance management strategy and help keep out malaria after elimination.<br><br>Could be applied through the private sector. More sustained reduction in vectorial capacity compared with other methods.<br><br>Effective against multiple vectors and vector-borne diseases.
Privacy	The device shall allow instillation of wind-blocking material for cold months and additional privacy.
Manufacturing considerations	Shall be capable of being mass produced and efficiently packaged.<br><br>Shall be produced by paid adult labourers under safe working conditions.
Transportation robustness	The device and packaging must pass MIL-STD-810G regarding travel over paved and un-paved roads.
