# Supplementary material for: New Prototype Screened Doors and Windows for Excluding Mosquitoes from Houses: A Pilot Study in Rural Gambia
Source: Am J Trop Med Hyg. 2018 Oct 22;99(6):1475–84. doi: 10.4269/ajtmh.18-0660 (PMC6283509; doi:10.4269/ajtmh.18-0660)
Supplement: Supplementary file 2 [file tpmd180660.SD2.pdf]

**Target Product Profile: Doors for Mosquito Exclusion**	
**Characteristic**	**Response**
Key assumptions	There is a growing market in Africa for modern houses.<br><br>Home-owners are investing in house improvement products.<br><br>Home owners will invest in better quality doors that prevent mosquito entry if they are competitive in price with existing products<br><br>Modern houses are associated with reduced malaria transmission in Africa<br><br>Screened doors will reduce mosquito house entry while increasing airflow indoors to keep the house cool<br><br>Increased comfort resulting from more air circulation will enhance mosquito net use.
Rationale	Doors that exclude mosquitoes from houses while increasing air flow (cooling the house) and providing security and privacy will be invested in by home owners. Minimising mosquito entry to houses will minimize multiple vector-borne diseases
Goal of the device	To exclude mosquitoes from entering houses through both screening/venting and self-closing mechanism while (1) maximizing air flow through the door, (2) providing security (lockable & robust to forced entry) and (3) privacy.
Target population	sub-Saharan Africa & potentially other tropical and sub-tropical countries
Setting	Rural and urban
Specifications	Shall meet ISO C3 corrosion standard.<br><br>Capable of withstanding "knife shear test" of a trimming knife is pulled x3 along screening with a force of up to 350N (35kg) and a constant downward pressure of 150N (15kg) for a distance of 250mm (https://crimsafe.com.au/testing/)<br><br>Ventilation provided by maximum pore size capable of excluding mosquito entry<br><br>For installation by semi-skilled workers.<br><br>Shall be ADA compliant i.e. a 90cm wheelchair can pass through the door.<br><br>Easily cleanable.<br><br>Meets water-resistant IPX-2 standard i.e. protecting against sprayed water when tilted up to 15

	degrees vertically (driving rain storm).<br>Should not weigh more than 50 Kg.<br>Miminum life of 10 yrs as determined by accelerated testing<br>Frame shall have the means to be securely fixed to the entry opening.
Operational price/ item	>$10/yr/door (assuming an effective live of >10 yrs – to be determined by accelerated lifecycle testing) (price point to be refined from market analyses of costs of present doors in Africa)
Compatibility	To be used with long-lasting insecticidal nets (increased air flow from screened doors should cool houses and minimize a significant reason why people do not sleep under LLINs (e.g., too hot)
Waste disposal	Should be recyclable. At end of life all materials must be disposable.
Drop requirement	n/a
Safety	Should be safe to users – should be designed to not cause injury under normal use. If the door is locked, shall allow egress from indoors i.e. a locked door has to be able to be unlocked from inside.
Benefit over existing methods	Contains no insecticides - use as an insecticide-resistance management strategy<br>Could be applied through the private sector (when near elimination, the public sector may be less willing to invest in malaria control).<br>More sustained reduction in vectorial capacity compared with other methods (longer effective life) - and reduce receptivity to malaria after elimination<br>Effective against multiple vectors and vector-borne diseases.
Privacy	The occupants shall not to be visible by people sitting or standing outside the house. The device shall allow instillation of wind-blocking material for cold months and additional privacy.
Manufacturing considerations	Shall be capable of being mass produced and efficiently packaged. Shall be produced by paid adult labourers under safe working conditions.
Transportation robustness	The device and packaging must pass MIL-STD-810G regarding travel over paved and un-paved roads.
